# Child Maltreatment Reporting Practices by a Person Most Knowledgeable for Children and Youth: A Rapid Scoping Review

**DOI:** 10.3390/ijerph192416481

**Published:** 2022-12-08

**Authors:** Ashley Stewart-Tufescu, Isabel Garces-Davila, Samantha Salmon, Katerina V. Pappas, Julie-Anne McCarthy, Tamara Taillieu, Sonya Gill, Tracie O. Afifi

**Affiliations:** 1 Faculty of Social Work and Children’s Hospital Research Institute of Manitoba, University of Manitoba, Winnipeg, MB R3T 2N2, Canada; 2 Department of Community Health Sciences, University of Manitoba, Winnipeg, MB R3E 0W2, Canada; 3 Departments of Community Health Sciences and Psychiatry, Children’s Hospital Research Institute of Manitoba, University of Manitoba, Winnipeg, MB R3E 0W2, Canada

**Keywords:** child maltreatment, person most knowledgeable, parents, reporting practices, population surveys, concordance, discordance, children, youth

## Abstract

Child maltreatment is a global public health and child rights crisis made worse by the ongoing COVID-19 pandemic. While understanding the breadth of the child maltreatment crisis is foundational to informing prevention and response efforts, determining accurate estimates of child maltreatment remains challenging. Alternative informants (parents, caregivers, a Person Most Knowledgeable—PMK) are often tasked with reporting on children’s maltreatment experiences in surveys to mitigate concerns associated with reporting child maltreatment. The overall purpose of this study was to examine child maltreatment reporting practices in surveys by PMKs for children and youth. The research question is: “What is the nature of the evidence of child maltreatment reporting practices in general population surveys by PMKs for children and youth?” A rapid scoping review was conducted to achieve the study’s purpose. A search strategy was conducted in nine databases (e.g., MEDLINE, EBSCO, Scopus, Global Health, ProQuest). The findings from this review indicate that most studies involved PMK informants (i.e., maternal caregivers), included representative samples from primarily Western contexts, and utilized validated measures to assess child maltreatment. Half of the studies assessed involved multi-informant reports, including the PMKs and child/youth. Overall, the congruence between PMK-reported and child/youth-reported child maltreatment experiences was low-to-fair/moderate, and children/youth reported more maltreatment than the PMKs.

## 1. Introduction

Child maltreatment is an urgent global public health and child rights crisis [1,2,3] made worse by the ongoing COVID-19 pandemic in countries worldwide [1]. Child maltreatment, including experiences of physical abuse, emotional abuse (psychological violence), sexual abuse, physical neglect, emotional neglect, and exposure to intimate partner violence (IPV) [2], is associated with numerous detrimental health, social, educational, and economic consequences in childhood and adulthood [3,4,5]. Specific to health and well-being, child maltreatment is associated with leading causes of morbidity and mortality among young adults, including substance use and misuse, self-harming behaviours and suicidality, and risk-taking behaviours and violence [6]. Considering the significant and widespread burden of child maltreatment during childhood and throughout the life course, further surveillance and research are warranted to understand better the scope and impact of this health and social issue. Findings from such activities will help inform the development and implementation of evidence-based public health interventions and policies to address child maltreatment [7].

While understanding the breadth of the child maltreatment crisis is foundational to informing prevention and response efforts, determining accurate estimates of the occurrences of child maltreatment is complex and challenging due to the “hidden” nature of the experience. Global prevalence estimates vary significantly due to methodological differences in the definition, measurement, temporality, and geography examined and differences based on the informant reporting [8]. A series of meta-analyses conducted in 2016 estimated that nearly 1 billion children or 50% of the children in the world between 2 and 17 years of age experienced physical, sexual, and emotional abuse in a past year [9], while other estimates of child maltreatment range from 4% to 54% [8]. Additional barriers associated with estimating the prevalence of child maltreatment include the availability of resources to conduct population-representative surveys that assess these experiences, competing interests challenging the inclusion of child maltreatment measures in other broader health-focused surveys, ethical and privacy concerns, access to respondents, and reliability and confidence of responses provided [10].

Population-based surveys are a common approach used to determine estimates of child maltreatment and its associated risks from a particular context and temporality and create opportunities for international comparisons. Respondents to such surveys vary considerably and depend on several characteristics and factors, including the child’s age, developmental stage, cognitive capacity to consent, and access to participate in such opportunities [11]. Self-reporting experiences of child maltreatment is recommended [12,13,14], and surveying children and youth directly during childhood or in early adulthood may limit concerns of recall bias and stability of reported child maltreatment experiences over time (i.e., differences between prospective and retrospective measures) [15]. Surveying children and youth directly also allows them to assert their agency and actualize their right to participate in matters that concern them [16], although it is important to note that concerns have been raised in the literature about the quality of data garnered from self-report surveys regarding younger children (less than 12 years of age) compared to adolescents [14,17]. Previous research has suggested that children under the age of 10 years may not be reliable reporters about maltreatment experiences due to their developmental stage compared to older children [18,19]. Other research recommends using interviewers to administer measures for children younger than 10 years of age [14]. Recent research has also found that most child/adolescent self-reported measures are not widely used nor have been evaluated for psychometric rigour, providing little evidence regarding the validity and reliability of most measures with a few exceptions [14].

To mitigate some of these concerns associated with the reliability and feasibility of children’s self-reporting, alternative informants or proxies are often tasked with reporting on children’s maltreatment experiences in surveys. The term Person Most Knowledgeable (PMK) is commonly used in the literature to describe a parent or caregiver who can report on a child’s experiences. Notably, the term Person Most Knowledgeable can be defined in several ways with different assumptions based on the child’s age, knowledge about the child’s everyday life, parenting styles (e.g., authoritative, neglectful), household composition (e.g., divorced parents), parental physical and mental health status, family financial situation, relationship with index child, among others. However, this informant approach is not without its unique concerns. Important questions have been raised about the quality of data generated from PMKs, including whether they have the relevant knowledge to report on a child’s experiences and the potential for under-reporting such incidents should the PMK be the perpetrator of the maltreatment being assessed. While these concerns are important and worthy of further investigation, reporting bias concerns are not localized to child maltreatment research. For example, statistical corrections have been developed to adjust for well-known discrepancies in self-reported height and weight measurements in health-related research. These correction factors have minimized reporting biases to varying degrees of success in this field of study [20,21,22,23]. In summary, there is still much to understand about child maltreatment reporting practices by alternative informants (i.e., PMKs) for children and youth and a need to examine opportunities to mitigate notable limitations to using this approach for child maltreatment surveillance and research.

The overall purpose of this research was to examine child maltreatment reporting practices by PMKs for children and youth in surveys. The broad research question guiding this work is: “What is the nature of the evidence of child maltreatment reporting practices in surveys by PMKs for children and youth?” The specific objectives of this research were to: (1) identify published, peer-reviewed studies that collected survey data about children’s and youths’ experiences of child maltreatment from a PMK; (2) examine the methods and measures utilized in studies to document experiences of child maltreatment, including physical abuse, emotional abuse, sexual abuse, physical neglect, emotional neglect, and exposure to IPV, using a PMK informant; (3) assess if the methods and measures reported by the PMK differed by the age of the target child (0–18 years); (4) summarize best practices for asking PMKs about child maltreatment experiences for children and youth in surveys; and (5) recommend feasible and evidence-based strategies to address identified limitations associated with using PMKs to report on child maltreatment experiences of children and youth in surveys.

## 2. Materials and Methods

A rapid scoping review was conducted to achieve the study’s purpose and objectives. It was implemented following the World Health Organization (WHO) Rapid Review Guide and the Joanna Briggs Institute 2020 Guide to conducting scoping reviews, which extends the methodological work of Arksey and O’Malley (2005) [24] and Levac, Colquhoun, and O’Brien (2010) [25]. The results of this review are reported using the Preferred Reporting Items for Systematic Reviews and Meta-Analyses (PRISMA-ScR) modified for scoping reviews [26].

### 2.1. Search Strategy and Information Sources

To identify the potentially relevant studies for this review, we consulted with an experienced medical librarian (O. Dingwall) from the University of Manitoba. Three team members (A.S.T., I.G.D., and K.V.P.) created the search strategy with guidance from O. Dingwall. Search terms included (“child maltreatment” OR “child abuse”), (“concordance” OR “discordance” OR “parental informant,” OR “Person Most Knowledgeable” OR “reporter agreement”).

Two team members (I.G.D. and K.V.P.) independently ran the search strategies in select databases. In total, 11 bibliographic databases were searched and yielded a total of 1504 citations: PubMed (*n* = 560), Medline (Ovid, *n* = 355), PsycInfo (Ovid, *n* = 19), EMBASE (Ovid, *n* = 117), Global Health (Ovid, *n* = 146), CINAHL (EBSCO, *n* = 149), EconLit (EBSCO, *n* = 20), Web of Science (*n* = 54), Cochrane (*n* = 9), ERIC (*n* = 63), and Scopus (*n* = 1). The final search strategy executed for MEDLINE was performed on 13 February 2022 (see Supplementary Material Table S1 for the detailed strategy).

### 2.2. Eligibility Criteria

For studies to be included in the review, papers needed to align with the pre-defined elements of the Population or Participants/Concept/Context framework (PCC). The Population (Participant) element included a PMK older than 18 years of age, any sex or gender, parent, caregiver/guardian, or teacher reporting on children’s or youths’ maltreatment experiences. The Concept element included the phenomena of interest: forms of child maltreatment including physical abuse, emotional abuse, sexual abuse, physical neglect, emotional neglect, or exposure to IPV, and reporting/data collection methods, including population-level samples, surveys, and questionnaires. The Context element involved studies published in English and French, conducted in both developing and developed regions of the world, and primarily quantitative research studies of various study designs.

Generally, for a full-text article included in the review for data extraction purposes, the study needed to clearly describe a PMK reporting children’s or youths’ experiences of one or more types of child maltreatment in a survey or using a questionnaire [26,27]. Included studies were published between 2000 and 2022 (January), were written in either English or French, described the type of child maltreatment assessed, how child maltreatment was measured and conceptualized, and a description of the PMK and child characteristics. Articles were excluded if they did not align with the PCC framework; only examined Adverse Childhood Experiences (ACEs) or household dysfunction without specific mention of forms of child maltreatment; only examined corporal punishment; only examined peer violence or community violence; were studies that utilized primarily qualitative methods, or were commentaries, case reports, conference abstracts or grey literature such as reports from non-governmental organizations.

### 2.3. Selection of Sources of Evidence

Before any information was extracted from the identified studies, the team participated in a rigorous screening training process to ensure consistency among the reviewers. Two reviewers independently screened every title/abstract derived from the search strategy as either “Yes,” “No,” or “Maybe.” Any disagreements between the reviewers’ ratings were decided by the first and second authors, A.S.T. and I.G.D. All pairs of reviewers achieved a proportionate agreement of 80% or greater before selecting sources of evidence in this review.

Similarly, at the full-text screening stage, each full-text article was independently reviewed by two reviewers and screened as either “Include” or “Exclude.” The final decision to include or exclude the paper and the reason for exclusion were determined between A.S.T. and I.G.D. Inter-rater reliability between A.S.T. and I.G.D. was kappa = 1.0, indicating perfect agreement for full-text review.

### 2.4. PRISMA Results

**Figure 1:**
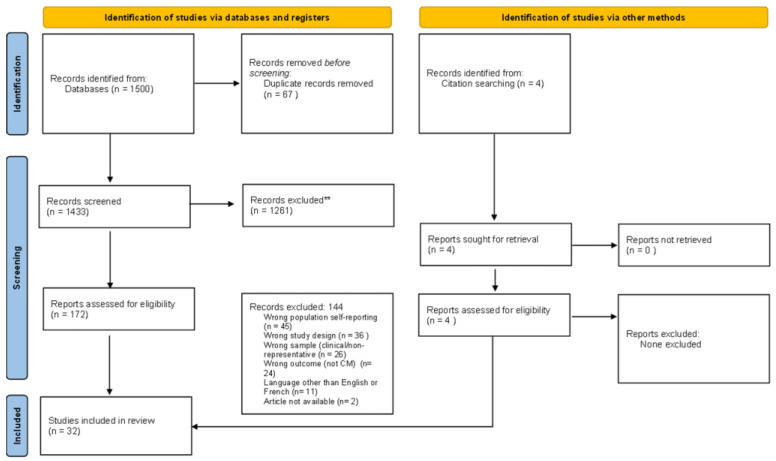
PRISMA Flow Chart. ** All records were screened by A.S.T. and I.G.D., no automation tools were used to exclude studies.

Figure 1 displays the PRISMA flow chart showing that 1504 citations were identified through database and citation chaining searching and imported for screening.

### 2.5. Data Extraction

The team developed a standardized data extraction form to determine which variables to extract. Before extracting data, the template was pilot tested by A.S.T. and I.G.D. Only one reviewer (A.S.T., I.G.D., or J.M.) completed data extraction for each study. Owing to the volume of data extracted from the studies, four independent coding keys were created (coding keys are available upon request to the authors). Table 1 presents an overview of the data extraction template.

## 3. Results

### 3.1. Objective 1

Identification and sample characteristics of included published, peer-reviewed studies that collected survey data about children’s and youths’ experiences of child maltreatment from a PMK.

After screening 1504 titles and abstracts and 172 full-text documents, data were extracted from 32 peer-reviewed articles.

#### 3.1.1. Characteristics of Included Studies

All included studies were published in the past 15 years (since 2007), and the percent distribution of the year of publications showed that nearly half of the included studies (*n* = 15; 46.9%) were published between 2015 and 2019 (refer to Table 2. Document characteristics). More than half of the studies were conducted in Western countries (*n* = 20; 62.5%), with the majority coming from the United States (*n* = 7; 21.9%). Three of the 32 studies were conducted in Canada (*n* = 3; 9.4%).

#### 3.1.2. Sample Size

The sample sizes for the included studies ranged from less than 1000 participants to over 2000 participants.

#### 3.1.3. Characteristics of Parents and Caregivers

Twenty studies (62.5%) in this rapid scoping review included data from parents and caregivers who identify as male or female, six (18.8%) reported data from females, and one study (3.1%) described paternal data [28]. The age ranges of parents and caregivers varied in the studies. Six studies (18.8%) included parents aged 15 to 60, while seven (21.9%) had parents aged 29 to 88. More details of parents and caregivers can be found in Table 3.

#### 3.1.4. Characteristics of Children and Youth

As indicated in Table 3, of the studies included in this review, 84% (*n* = 27) reported children and adolescents’ gender or sex as females and males. In contrast, 16% (*n* = 5) of the studies did not report the gender or sex of children and adolescents. Age ranges for children and youth varied in the included studies. Eleven studies (43.4%) reported ages between 8 and 18 years, while ten (31.3%) reported periods from birth to 18 years.

### 3.2. Objective 2

Examination of study design, methods and measures used to document experiences of child maltreatment, including physical abuse, emotional abuse, sexual abuse, physical and emotional neglect and exposure to IPV, using a PMK informant.

#### 3.2.1. Study Design

The studies included in this scoping review were descriptive and analytical More than half of the studies used an analytical cross-sectional design (*n* = 17; 53.1%) to compare reports of child maltreatment between children and parents. See Table 4 for information on the study design.

#### 3.2.2. Child Maltreatment Measurement

In more than 70% (*n* = 23) of the studies included, validated measures were used to assess child maltreatment experiences. These measures included The Conflict Tactic Scale: Parent–Child (CTS-PC), which was the most frequently reported measure to assess PMKs reporting of child and youth child maltreatment (*n* = 12; 37.5%), followed by the Juvenile Victimization Questionnaire (JVQ; *n* = 6; 18.8%), and the ISPCAN Child Abuse Screening Tool for Parent (ICAST-P; *n* = 4; 12.5%; see Table 4 for additional details on the instruments).

#### 3.2.3. Types and Number of Child Maltreatment

The articles included in this review consisted of studies that examined singular and multiple experiences of child maltreatment for children and youth reported by a PMK. Physical abuse was the most common form of child maltreatment assessed in the included studies (*n* = 30; 93.8%), followed by emotional abuse (*n* = 26; 81.3%), physical neglect (*n* = 17; 53.1%), emotional neglect (*n* = 16; 50.0%), and sexual abuse (*n* = 15; 46.9%). Exposure to IPV was the least common form of maltreatment examined in the studies (*n* = 9; 28.1%). The number of maltreatment experiences measured ranged from one type (15.6%) to all six (12.5%) types. Physical abuse only was examined in three studies (9.3%), and sexual abuse only and IPV only were examined in one study (3.13%; see Table 4 for additional details on the types and number of maltreatment experiences assessed in the included studies).

### 3.3. Objective 3

Assessment of the methods and measures reported by the PMK depending on the age of the target child (0–18 years).

#### Methods and Measures to Assess Child Maltreatment

As described in Table 4, 14 studies (43.7%) collected child maltreatment information from children and youth with face-to-face interviews and trained research assistants in a home or school setting. Three studies used more than one questionnaire to assess maltreatment [31,32,33].

Of those studies that collected child maltreatment information with face-to-face interviews, 10 (31.2%) used the Parent–Child Conflict Tactics Scale (CTS-PC), while five (15.6%) used the Juvenile Victimization Questionnaire (JVQ), and one (3.0%) used the International Child Abuse Screening Tool for Parent (ICAST-P). Of those studies that gathered child maltreatment data with computer-assisted methodologies, one study (3.1%) used the Childhood Experiences of Violence Questionnaire (CEVQ), another (*n* = 1; 3.1%) used the Parent–Child Conflict Tactics Scale (CTS-PC) and one study (*n* = 1; 3.1%) the International Child Abuse Screening Tool for Parent (ICAST-P; see Table 5 for more details on child maltreatment instruments according to children and adolescents age).

### 3.4. Objective 4

Summarize the best practices for asking PMKs to report on child maltreatment experiences of children and youth in surveys.

#### 3.4.1. Concordance between Informants

In this review, nearly all included studies that examined the agreement between multi-informants reported low to fair/moderate levels of agreement. Concordance rates/levels of agreement varied by the subtypes of child maltreatment assessed (e.g., physical abuse vs. sexual abuse), the severity of the maltreatment, past-year versus lifetime assessment, and characteristics of the informants (e.g., gender of PMK and child, age of the child/youth).

#### 3.4.2. Types of Child Maltreatment and Informant Agreement

Three studies reported differing levels of agreement based on the child maltreatment types examined (e.g., physical or sexual abuse). One study examined concordance rates of physical abuse between three dyads (child–caregiver, caregiver–caseworkers, and child–caseworker) and reported low concordance between informants, indicating children reported higher rates of maltreatment experiences compared to their caregivers and caseworkers [32]. Overall, concordance between dyads (i.e., mother–child, father–child, and mother–father) and subtypes of child maltreatment (e.g., physical abuse, emotional abuse, physical neglect and emotional neglect) was found to be low to moderate (intraclass correlation coefficients <0.35).

#### 3.4.3. Concordance Assessments between Informants and Type of Child Maltreatment Reported

As described in Table 6, most of the studies (*n* = 24; 75%) included in this review did not report concordance measures between informants (e.g., Intraclass Correlation Coefficient). Of the studies that did report concordance measures, two of the studies (6.3%) used the Intraclass Correlation Coefficient (ICC) and examined physical abuse, emotional abuse, physical neglect, and emotional neglect [31,34]. Similarly, two studies (6.3%) reported Cohen’s kappa coefficients, including physical abuse, emotional abuse, and physical and emotional neglect [35,36].

#### 3.4.4. Informant Agreement and PMK Characteristics

Other studies examined the role of parental gender on the congruence of child and parent–child maltreatment reporting practices. Regardless of parent gender, parents were less likely to report more severe experiences of physical violence and more likely to report more minor incidents of physical assault, psychological aggression, and neglect compared to child informants [35]. Similarly, there was low agreement between parent–child and mother–father dyads reporting physical child abuse, where children reported more child maltreatment than the PMK informant. Minimal gender differences were noted for child maltreatment experiences other than physical abuse; mother–girl child dyads and mother–boy child dyads had better agreement than father–child dyads specific to physical abuse only [37].

### 3.5. Objective 5

Recommend feasible and evidence-based strategies to address identified limitations associated with using PMKs to report on child maltreatment experiences of children and youth in surveys.

Most studies (68.8%) noted limitations and concerns about PMK informants reporting on children and youths’ maltreatment experiences in surveys. Three studies [29,35,38] stated that parents and caregivers under-reported severe forms of child maltreatment (e.g., two or more types of abuse). Two studies indicated that parents over-reported psychological and less severe forms of physical abuse [29,35]. For example, Chan (2012) [35] suggests that children reported more severe forms of physical assault and neglect by fathers. Of the studies that reported limitations/concerns, 12 (54.5%) noted that PMKs reporting child maltreatment might be affected by bias. Of these, eight studies indicated recall, denial, distortion, and “wish” biases (i.e., a tendency on the part of the participant for the desired outcome) [31,33,34,39,40,41,42,43], and three stated social desirability bias as the primary concern [41,43,44]. Table 7 displays the limitations reported.

## 4. Discussion

This rapid scoping review aimed to examine the nature of the evidence of child maltreatment reporting practices by PMKs for children and youth in surveys. This review examined 32 studies using a PMK informant to report on children’s and youths’ maltreatment experiences utilizing a survey. The data extracted from the studies addressed the overall research question and the five research objectives, including identifying relevant studies and study characteristics; examining the study design, methods and measures used to document experiences of child maltreatment using a PMK informant; assessing the methods and measures based on the ages of the children/youth studied; considering the best practice of using a PMK informant to report these experiences in surveys; and identifying limitations with the approach and recommending feasible and evidence-based strategies to mitigate identified limitations. Although this review maps out the evidence on child maltreatment reporting practices, it is relevant to note the results are limited by the available published peer-reviewed evidence (predominantly from Western countries) with populations sharing cultural proximities, including language and views on child maltreatment (e.g., U.S. and Canada). Thus, the utility of this review is to provide an overview of research from peer-reviewed and published studies that may guide future research and public health surveillance strategies regarding the maltreatment experiences of children and youth.

To elaborate on key findings from this review, it was found that more than half of the studies (62.5%) were conducted in Western countries, utilized standardized measures with established reliability and validity with representative samples, and involved a PMK informant that was a maternal-caregiver figure in relation to the index child (75%). Related to an assessment of child maltreatment experiences, few studies included in this review examined all maltreatment types, nor the severity, frequency, or duration of such incidents. Notably, only four of the studies assessed all six maltreatment types, and exposure to IPV was the type of maltreatment least likely to be examined. Given the reliance on the maternal caregiver assuming the role of the PMK informant and evidence suggesting that concordance is better between mother–child than father–child dyads for reporting physical abuse [37], further research should examine the role of paternal caregivers as the PMK informant on child maltreatment reporting practices. Together these findings highlight some critical gaps in assessing child maltreatment experiences by a PMK informant in a survey, with specific consideration being how best to define and identify a PMK informant for children in nationally representative surveys recognizing the diverse and ever-changing portrait of families in Canada [45].

One of the most critical findings from this review is the discrepancies between PMK informants’ reports of children’s maltreatment experiences and reports from children themselves and the noted concerns with PMK biases. Of the studies that included a multi-informant assessment (46.5%), the agreement was generally characterized as low to fair/moderate. Based on the studies examined in this review, children/youth tend to report more experiences of child maltreatment compared to those reported by the PMK. Moreover, PMKs report less severe forms of physical abuse than children/youth reports. Interestingly, these findings are similar to results from a recent systematic review of informant discrepancies in child maltreatment, which examined youth reports, caregiver reports (residential care staff, parents), and child protective services case reports and found agreement among all types of maltreatment was generally poor [46]. The systematic review found that youth reported more maltreatment experiences than the caregivers for physical, emotional, and sexual abuse, with differences ranging from 0.5% to 37.5% depending on the type of abuse assessed, except for neglect (for youth–caregiver reports but not for youth–case file reports). Notably, youth reports of all maltreatment types were more than child maltreatment experiences documented in case files.

Lack of concordance was the most frequently stated limitation/concern in studies that noted challenges with the PMK approach to assessing children and youth maltreatment experiences in surveys. In our scoping review, the lack of concordance between PMK and child/youth reports may reflect PMK reporting biases. One study [35] indicated that adolescents also tend to under- and over-report maltreatment experiences; after further analysis, the author stressed that a higher level of parental social desirability is associated with a tendency to under-report maltreatment experiences in adolescents. Whereas Naicker and colleagues (2017) [30] suggest that discrepancies found in their longitudinal study between adolescent-reported experiences of child maltreatment, retrospective reports in young adulthood and prospective caregiver reports might be partially explained due to development, maturation, and differences in memory, cognition, and emotional state, as well as methodological concerns given that caregivers did not report on the youths’ experiences of child maltreatment during adolescence (ages 15–18), a time of life when young people often report physical and sexual abuse experiences. Additional prospective and longitudinal studies are warranted to understand better these crucial differences in child maltreatment experiences reported by informants and how these reports may change over time.

While the results of this scoping review indicate discrepancies in reports of child maltreatment experiences between parents and children, these findings should be interpreted with caution and not be used to justify the exclusion of PMKs (parents and other caregivers) from reporting on child maltreatment experiences of children and youth, especially given the importance of the PMK in reporting child maltreatment experiences of younger children in surveys (e.g., under ten years of age; [14,47]). Instead, these findings indicate that future research and surveillance initiatives should ensure that PMK-based reporting is sensitive to confidentiality issues and aware of the potential for social desirability and reporting biases. In an effort to address some of the limitations and concerns with PMKs reporting on children’s and youths’ child maltreatment experiences in surveys, future methods should emphasize self-administered formats rather than face-to-face interviews; include audio- and computer-assisted technologies that allow for enhanced anonymity and confidentiality; provide PMK respondents with easily accessible counselling and other supports, should they find the experience of completing the survey distressing; and ensure that surveyed personnel engage with PMK-informants with sensitivity and non-judgement [47]. Furthermore, future research and surveillance initiatives may benefit from implementing a multi-informant approach that directly includes PMKs and children/youth informants. As noted in previous research, a combination of survey reporters (i.e., caregivers, adolescent self-reports, and sentinel reports–child-serving professionals/care reports) is optimal to establish the prevalence of child maltreatment experiences [48].

The necessity for data to accurately understand the scope of child maltreatment in Canada was recently identified as a critical child rights issue by the United Nations Committee on the Rights of the Child (CRC/C/CAN/CO/5-6, 2022). In the Committee’s concluding observations of the Government of Canada’s fifth and sixth reports on compliance with the United Nations Convention on the Rights of the Child, the Committee recommended that the Government of Canada improve its data collection system at the federal level. Doing so will allow for (1) nationwide comprehensive monitoring of the rights of children with data disaggregated by sociodemographic characteristics, focusing on children in situations of vulnerability; and (2) to ensure that data and indicators on children’s rights include all children below the age of 18 years and are shared with ministries concerned and used for the development, monitoring, and evaluation of policies and programmes to implement children’s rights in Canada. Engaging with children and youth directly in research to understand their experiences of child maltreatment will be an essential contribution to addressing significant child rights concerns in Canada.

Given the conflicting reports of child maltreatment experiences between parents and children, Chan (2012) [35] suggests using simultaneous reports of PMKs and children to accurately assess prevalence estimates of child maltreatment and interventions for those at risk. In contrast to some of the results mentioned above, the findings from this scoping review also indicate that agreement on emotional abuse was higher between adolescents and parents compared to children and parents. This finding suggests that future research should integrate age-appropriate (e.g., pictures, language, length of questionnaires, types of questions) child maltreatment instruments and subscales highlighting each type of maltreatment and the age of participants. Many of these recommendations were also noted in a recent systematic review of psychometric properties of child and adolescent violence self-reporting measures [14]. Based on the results from the noted systematic review by Meinck and colleagues (2022) [14], however, some suggested approaches (e.g., using pictures and reading level adjustment) may not be feasible or easily implemented in nationally representative surveys [14]. A methodological report using the Childhood Trauma Questionnaire—Short Form (CTQ-SF) sheds light on the validity and reliability of asking youth to report their childhood maltreatment experiences [49]. Hagborg et al. (2022) [49] used the CTQ-SF to examine the instrument’s acceptability and psychometric properties in adolescents. The findings suggest the CTQ-SF presents test–retest stability that increased over time in their study, thus indicating that adolescents provide more reliable answers from the age of 14 than earlier [49]. The authors report that the CTQ-SF proved to be a valid instrument for measuring child maltreatment when administered at ages 14–15 than in earlier periods [49]. More methodological studies are needed, and future research should also focus on methods and measures that are safe and developmentally sensitive for child and youth informants.

Another opportunity to improve understanding of child maltreatment experiences using a PMK informant in surveys is via the methods and measures. As noted above, there is a limited assessment of the severity, frequency, and duration of child maltreatment experiences. Future research should address these issues while considering respondent burden, including time for completion, privacy and sensitivity of questions, and availability of resources to facilitate surveys. A rapid review of child maltreatment measures reported in the *World Health Organization Practical Handbook for Measuring and Monitoring Child Maltreatment in European Countries* [47] offers insights worth considering for future survey-based research in the Canadian context. The authors recommend that research incorporate child maltreatment-specific questionnaires available at no cost, examine all maltreatment types and dimensions, are tested and used in different contexts (to allow for comparisons), and have reliable and valid data. Notably, the authors suggest that when concerns associated with funding, time for completion, or questionnaire space in other national surveys arise, efforts should be made to include a more concise yet comprehensive measure of maltreatment (e.g., Short Child Maltreatment Questionnaire (SCMQ) [47]). Further research should focus on formulating and testing measures and methods to provide a more comprehensive understanding of child maltreatment experiences assessed via surveys while balancing respondent burden among multiple informants here in Canada.

This scoping review is not without limitations. First, our results are limited by the availability of evidence from studies published primarily in English. This could have affected the study’s overall view of parenting reporting practices by omitting evidence from other countries and contexts where English or French is not the primary language used for knowledge dissemination. Additionally, the lack of published evidence from lower- and middle-income countries (LMIC) could have affected the conclusions of this study due to contextual and cultural differences. Owing to the available research examined, the results from this scoping review largely did not capture parental attitudes and views towards child maltreatment from LMIC, which might be different from those in high-income countries, such as the United States. Thus, the findings from this study may not be applicable to research conducted in LMIC. Future research should explore the child maltreatment reporting practices for children and youth by a PMK in studies conducted in settings outside of North America and Europe and published in languages other than English.

Second, most studies did not include detailed descriptions of the instrument to measure child maltreatment, exclusion of subscales, or adaptation of items, and how these considerations could have affected concordance reports between parents and children. Third, the studies included provided limited information about the survey processes. Most studies included scarce information on data collection procedures for parents and children/youth. Fourth, this scoping review examined different child maltreatment reports from PMKs. Studies that examined ACEs that included child maltreatment and reports of household challenges were not included, nor were studies that focused solely on corporal punishment. Fifth, although this study maps out the literature on the strengths and limitations of asking parents and caregivers to report on child maltreatment, scoping reviews do not include an analytical component as part of their methodology. A recent systematic review and meta-analysis noted that the discrepancy between parents’ and youths’ reports of child maltreatment varies by the type of maltreatment examined and the severity of the experience [46]. Thus, a meta-analysis was not conducted to examine the directionality and magnitude of concordance and discordance reports of child maltreatment between parents and youth across studies.

## 5. Conclusions

This research is the first rapid scoping review of child maltreatment reporting practices in surveys by a PMK for children and youth. In this review, the congruence between PMK-reported and child/youth-reported child maltreatment experiences was low-to-fair/moderate. Children and youth reported more maltreatment compared to the PMKs. Combined expertise and efforts among researchers, decision-makers, and clinicians could help identify the knowledge gaps and needs to incorporate survey methods that could be implemented to improve data and strategies to enhance child maltreatment measurement in nationally representative surveys. In summary, without accurate identification of child maltreatment experiences to understand the scope of this public health and child rights crisis, it will remain a challenge to develop, implement, and adequately resource child maltreatment prevention and response strategies and policies.

## Figures and Tables

**Table 1:** Data extraction categories.

Study/Context Characteristics	Concept Characteristics: Design and Measurement	Concept Characteristics: Informant Report Indicators	Population Characteristics
Author and Year Title Funding sources Study aim Geographical region(s)	Study design Method of data collection Sampling strategy Child maltreatment instrument Child maltreatment reported Conceptualization of child maltreatment	Validity and reliability of data Concordance measure between informants Details about concordance reports Details about response bias/concerns with PMK reporting	Sample size—number of PMK participants Age of PMK Gender or sex of PMK PMK relationship to the index child Sample size—number of child/youth PMK reported on Age ranges of child/youth Gender or sex of child/youth

**Table 2:** Document characteristics.

		Count (%) *n* = 32
Year of publication	<2010	1 (3.1)
	2010–2014	6 (18.8)
	2015–2019	15 (46.9)
	2020–2022	10 (31.3)
Geographical region	Africa	2 (6.3)
	Asia	7 (21.9)
	Australia and New Zealand	1 (3.1)
	Europe	9 (28.1)
	North America	10 (31.3)
	South America	1 (3.1)
	Multiple countries	2 (6.3)
Funding source type	Public-sponsored	24 (75)
	Industry-sponsored	0
	Non-sponsored	2 (6.3)
	Not reported	6 (18.8)
Journal discipline	Multidisciplinary	17 (53.1)
	Public Health/Epidemiology	4 (12.5)
	Psychology	3 (9.4)
	Social Work	2 (6.3)
	Violence	2 (6.3)
	Psychiatry	2 (6.3)
	Pediatrics	2 (6.3)

**Table 3:** Sample characteristics of included studies.

		Count (%) *n* = 32
Informant—PMK		
Age ^1^	15–60	6 (18.8)
	20–67	5 (15.6)
	29–88	7 (21.9)
	53–88/21–68	1 (3.1)
	Not reported	13 (40.6)
Gender or sex	Females	6 (18.8)
	Males	1 (3.1)
	Males and females	20 (62.5)
	Not reported	5 (15.6)
Relationship to the index child	Mother	3 (9.4)
	Father	1 (3.1)
	Mothers and fathers	24 (75.0)
	Caregiver not specified if mother or father	4 (12.5)
Informant—Child/Youth		
Age	0–18	10 (31.3)
	4–16	5 (15.6)
	6–12	3 (9.4)
	7–47	1 (3.1)
	8–18	11 (34.4)
	12–65	1 (3.1)
	5, 7, 11, 15, 18, 21, 22 ^2^	1 (3.1)
Gender or sex	Females and males	27 (84.0)
	Not reported	5 (16.0)
Sample size (PMK)	<1000	9 (28.1)
	1000–1999	7 (21.9)
	2000+	10 (31.3)
	Not reported	7 (21.9)
Sample size (child/youth)	<1000	11 (34.4)
	1000–1999	6 (18.8)
	2000+	14 (43.8)
	Not reported	2 (6.3)
Representative sample of children/youth	Yes	19 (59.4)
	No	4 (12.5)
	Unclear	9 (28.1)
Multiple informants	Yes	15 (46.9)
	No	11 (34.4)
	Not applicable	5 (15.6)
	Not reported	1 (3.1)

^1^ Includes study with mothers’ age range 15–49 (Akmatov et al., 2011 [29]). ^2^ Study included data from three age categories of children and youth (Naicker et al., 2017 [30]).

**Table 4:** Study design, methods, and measures to document experiences of child maltreatment.

		Count (%) *n* = 32
Design		
Study design	Cross-sectional study	17 (53.1)
	Longitudinal study	9 (28.1)
	Methods study	1 (3.1)
	Prevalence study	5 (15.6)
Method of data collection	Interview (face-to-face)	14 (43.7)
	Computer-assisted/Self-administered	13 (40.6)
	Research assistant over the phone	4 (12.5)
	Not reported	1 (3.1)
Measurement		
Instrument ^1^	Childhood Experiences of Violence Questionnaire (CEVQ)	2 (6.3)
	Parent–Child Conflict Tactics Scale (CTS-PC)	12 (37.5)
	Childhood Trauma Questionnaire (CTQ)	3 (9.4)
	International Child Abuse Screening Tool for Parent (ICAST-P)	4 (12.5)
	Juvenile Victimization Questionnaire (JVQ)	6 (18.8)
	Longitudinal Studies on Child Abuse and Neglect (LONGSCAN)	1 (3.1)
	Violence Exposure Scale (VEX-R)	1 (3.1)
	Maternal Interview on Child Maltreatment (MICM)	1 (3.1)
	Other ^2^	4 (12.5)
	Questionnaire developed for the study	2 (6.3)
Validated instrument ^3^	Yes	23 (71.9)
	No	1 (3.1)
	Unclear	2 (6.3)
	Not reported	6 (18.8)
Type of maltreatment ^1^	Physical abuse	30 (93.8)
	Sexual abuse	15 (46.9)
	Emotional abuse	26 (81.3)
	Physical neglect	17 (53.1)
	Emotional neglect	16 (50.0)
	Exposure to IPV	9 (28.1)
Number of maltreatment types measured	1	5 (15.6)
	2	6 (18.8)
	3	2 (6.3)
	4	9 (28.1)
	5	6 (18.8)
	6	4 (12.5)

^1^ Not mutually exclusive. ^2^ Life Incidence of Traumatic Events (LITE) questionnaire. Questionnaire based on the CDC/Kaiser Permanente ACE questionnaire. Questions derived from the Childhood Experiences of Violence Questionnaire (CEVQ) and the Childhood Trauma Questionnaire (CTQ). Children Health, Well-being, and Services Survey (LTH). ^3^ Validity and reliability measures.

**Table 5:** Child maltreatment measures and children/youth age ranges.

Child Maltreatment Instrument	Age Ranges Child/Adolescent Who Self-Reported Child Maltreatment	Count (%) *n* = 32
Parent–Child Conflict Tactics Scale (CTS-PC)	4–17 years	7 (22.0)
Juvenile Victimization Questionnaire (JVQ)	10–17 years	2 (6.3)
Questionnaire based on the CDC/Kaiser Permanente ACE questionnaire	21–22 years	1 (3.1)
Childhood Trauma Questionnaire (CTQ)	12–18 years	1 (3.1)
Maternal Interview on Child Maltreatment (MICM)	4–8 years	1 (3.1)
Life Incidence of Traumatic Events (LITE) questionnaire	12 years	1 (3.1)
Not reported		19 (59.4)

**Table 6:** Concordance measures between informants and type of child maltreatment reported.

	Type of CM	Physical Abuse, Emotional Abuse, Physical Neglect, Emotional Neglect Count (%)	Physical Abuse, Sexual Abuse, Physical Neglect, Emotional Neglect, and Exposure to IPV Count (%)	Physical Abuse Count (%)	Physical Abuse, Sexual Abuse, Emotional Abuse, Physical Neglect, Emotional Neglect Count (%) *n* = 32
Concordance Measure					
Cohen’s kappa		2 (6.3)	2 (6.3)	1 (3.1)	1 (3.1)
Intraclass Correlation Coefficient (ICC)		2 (6.3)	0	0	0
Not reported		0	24 (75)	0	0

**Table 7:** Limitations associated with using PMKs to report on child maltreatment experiences ^1^.

Limitations	Counts (%) *n* = 32
Under-reporting of CM, severe forms by PMK	3 (7.5)
Over-reporting of CM: psychological abuse and less severe forms of physical abuse	2 (5.0)
Reporter bias: information provided by the mothers over-estimated association; reports of IPV or CM biased by maternal mental health	1 (2.5)
Recall bias, denial, distortion, wish bias	8 (20.0)
Interpretation of CM different between mothers and fathers	1 (2.5)
Under- and over-reporting of CM by adolescents	1 (2.5)
Short questionnaire—less comprehensive, lack dynamic tools; maltreatment measures not exhaustive	3 (7.5)
Measures of CM severity	1 (2.5)
Social desirability bias	3 (7.5)
Disagreement between parent and child reports due to mental health–depression	1 (2.5)
Lack of concordance between parents, children/adolescents, caseworker	3 (7.5)
Selection bias	1 (2.5)
Culturally relevant CM items	1 (2.5)
Subjective views of caregivers and children in research assessment	1 (2.5)
Not reported: N/A	10 (25.0)

^1^ Each study reported several limitations.

## Data Availability

The data extraction coding keys generated from this study are available upon request. All other data are presented in the tables included in this paper.

## Supplementary Materials

The following supporting information can be downloaded at: https://www.mdpi.com/article/10.3390/ijerph192416481/s1, Table S1: Search Strategy.
