# Supplementary material for: Child Maltreatment Reporting Practices by a Person Most Knowledgeable for Children and Youth: A Rapid Scoping Review
Source: Int J Environ Res Public Health. 2022 Dec 8;19(24):16481. doi: 10.3390/ijerph192416481 (PMC9779242; doi:10.3390/ijerph192416481)
Supplement: Supplementary file 1 [file ijerph-19-16481-s001.zip › ijerph-2031955-SI.pdf]

Review

Child Maltreatment Reporting Practices by a Person Most Knowledgeable for Children and Youth: A Rapid Scoping Review

Ashley Stewart-Tufescu [1,*], Isabel Garces-Davila [2], Samantha Salmon [2], Katerina V. Pappas [2], Julie-Anne McCarthy [2], Tamara Taillieu [2], Sonya Gill [2] and Tracie O. Afifi [3]

Supplementary Material: Search Strategy from Medline
Database(s): Ovid MEDLINE(R) January 1, 2000 to February 13, 2022

Table S1: Search Strategy

#	Searches
1	Child maltreatment/
2	(Child* and (maltreat* or abus* or neglect*)).mp. [mp=title, abstract, original title, name of substance word, subject heading word, floating sub-heading word, keyword heading word, organism supplementary concept word, protocol supplementary concept word, rare disease supplementary concept word, unique identifier, synonyms]
3	(parent* or caregiver* or guardian* or "care provider" or person most knowledgeable).mp. [mp=title, abstract, original title, name of substance word, subject heading word, floating sub-heading word, keyword heading word, organism supplementary concept word, protocol supplementary concept word, rare disease supplementary concept word, unique identifier, synonyms]
4	(1 or 2) and 3
5	(survey* or questionnaire*).mp.
6	4 and 5
7	6
8	limit 7 to yr="2000 -Current"
9	(concordance or discordant or reporter agreement).mp.
10	4 and (5 or 9)
11	10
12	limit 11 to yr="2000 -Current"
